# Visceral adiposity indicators and cardiovascular risk in hemodialytic patients

**DOI:** 10.20945/2359-3997000000421

**Published:** 2021-11-11

**Authors:** Dejane de Almeida Melo, Alcione Miranda dos Santos, Elane Viana Hortegal Furtado, Ana Karina Teixeira França, Elisângela Milhomem dos Santos, Ilma Kruze Grande de Arruda, Tuane Rodrigues de Carvalho, Claudia Porto Sabino Pinho, Alcides da Silva Diniz, Maria da Conceição Chaves de Lemos

**Affiliations:** 1 Universidade Federal de Pernambuco Programa de Pós-graduação em Nutrição Recife PE Brasil Programa de Pós-graduação em Nutrição, Universidade Federal de Pernambuco, Recife, PE, Brasil; 2 Universidade Federal do Maranhão Departamento de Saúde Pública São Luís MA Brasil Departamento de Saúde Pública, Universidade Federal do Maranhão, São Luís, MA, Brasil; 3 Universidade Federal do Maranhão Departamento de Ciências Fisiológicas São Luís MA Brasil Departamento de Ciências Fisiológicas, Universidade Federal do Maranhão, São Luís, MA, Brasil; 4 Universidade Federal do Maranhão Departamento de Enfermagem São Luís MA Brasil Departamento de Enfermagem, Universidade Federal do Maranhão, São Luís, MA, Brasil; 5 Universidade Federal de Pernambuco Departamento de Nutrição Recife PE Brasil Departamento de Nutrição, Universidade Federal de Pernambuco, Recife, PE, Brasil; 6 Universidade Federal de Pernambuco Hospital das Clínicas de Pernambuco Recife PE Brasil Hospital das Clínicas de Pernambuco, Universidade Federal de Pernambuco, Recife, PE, Brasil

**Keywords:** Chronic renal insufficiency, cardiovascular diseases, adiposity

## Abstract

**Objective:**

Cardiovascular diseases represent the main cause of death in chronic kidney disease (CKD). We aimed to evaluate the prevalence and association of the hypertriglyceridemia-waist phenotype (HWP) and visceral adiposity index (VAI) with cardiometabolic risk factors (CR) in patients with CKD on hemodialysis (HD).

**Materials and methods:**

The study is based on a cross-sectional design with 265 HD patients in two cities in northeastern Brazil. The VAI was calculated considering the variables body mass index (BMI), waist circumference (WC), triglycerides (TG) and high density lipoprotein cholesterol (HDL-c). HWP was defined as the concomitant elevation of WC and TG. The Poisson Regression Model with robust variance estimation was adjusted considering a hierarchical approach for explanatory variables. Prevalence ratios (PR) were also estimated. The level of significance adopted was 5%.

**Results:**

In our study HWP and VAI prevalence’s were 29.82% and 58.49%, respectively. In the final model, there was an association between VAI and female gender (PR = 1.46; p < 0.0001) and high body fat (% BF) (PR = 1.33; p < 0.0019). HWP was associated with females (PR = 1.80; p = 0.002), alcohol consumption (PR = 1.58; p = 0.033), obesity (PR = 1.89; p = 0.0001), high %BF (PR = 1.76; p = 0.012) and reduced HDL-c (PR = 1.48; p = 0.035).

**Conclusion:**

The HWP stood out as the association with more CR factors, representing a promising method for tracking cardiometabolic risk in HD patients, mainly female.

## INTRODUCTION

Chronic kidney disease (CKD) is a public health problem defined as an abnormality of kidney structure or function present for more than three months, with health implications (1). Among patients with CKD, cardiovascular disease (CVD) is the main cause of death at any stage of the disease (2).

Although traditional cardiovascular risk factors (CR) cannot alone explain the high risk of CVD presented by patients on hemodialysis (HD), they seem to be highly predictive of this nosological entity (3). In this context, the investigation of the role of visceral adiposity has gained prominence since it has been associated with metabolic abnormalities in patients under dialysis treatment (4).

Predictive equations seek to find a practical and accessible marker based on indirect measurements of visceral adiposity, among which are visceral adiposity index (VAI) and the hypertriglyceridemia-waist phenotype (HWP) as identification tools of visceral adipose dysfunction and atherogenic metabolic triad (5-6).

VAI has been reported as an indicator associated with long-term cardiovascular outcomes. All-cause mortality (7) and metabolic syndrome (8). HWP manifests as worse carotid atherosclerosis in patients with CKD (9).

Thus, taking into account the impacts of visceral adiposity on cardiovascular events frequently found in the HD scenario, in addition to the scarcity of studies assessing such indexes in the Brazilian HD population and, above all, in the northeastern region of Brazil, where resources are scarce, the present study aims to assess HWP prevalence and visceral adipose dysfunction according to VAI and to analyze its association with traditional CR factors in patients with CKD on HD.

## MATERIALS AND METHODS

### Study design

This is a cross-sectional study that performs the analysis of two databases from two studies that evaluated patients with CKD undergoing dialysis at two capitals in the northeast of Brazil (São Luís, MA, and Recife PE) from January to December 2016 (10-11).

### Ethical aspects

The present study obtained approval from the Research Ethics Committee involving Human Beings of the *Hospital das Clínicas* of the Federal University of Pernambuco (CAAE 25657819.0.0000.8807) in accordance with the Resolution no. 466/2012. All study participants signed the informed consent.

### Sample, inclusion and exclusion criteria

Clarifications regarding the population and inclusion criteria for the previous studies carried out in the cities of São Luís and Recife have been reported in previous studies (10-11). In brief, patients were regularly registered in the dialysis program undergoing renal replacement therapy for at least three months and for least three-hour dialysis sessions of both genders, aged 18 years or over, who signed the Informed Consent. The following category of patients were not included: pregnant, amputated, those with neurological diseases or stroke sequelae, autoimmune and infectious diseases, cancer, and acquired immunodeficiency syndrome. Volunteers were recruited during HD sessions held in 2016 at São Luís (MA) by stratified random sampling and at Recife (PE) by convenience. At that time, the objectives, risks, benefits, procedures adopted in the research, and study eligibility criteria were explained. Eligible patients obtained express authorization to participate in the study by signing the Informed Consent. All evaluated patients who had fulfilled the interest variables for the present study were simultaneously included.

### Sample calculation

For those studies, the sample was calculated a posteriori using the software OpenEpi, version 3.01, considering the sum of HD patient populations of the seven evaluated centers, the prevalence reported in the literature of 24.6% (12), and adopting a 95% level of significance and a 80% test power. Thus, the minimum sample required would be 237 patients.

The sum of databases from both studies included data from 468 patients. Since only 265 patients fulfill the prerequisite of having all interest variables, these comprised the study sample.

### Sociodemographic and lifestyle variables

The following sociodemographic characteristics were analyzed: age, gender, self-reported color (white, black, brown, and others), monthly family income (described in minimum wages – MW – for 2016), education (dichotomized in up to nine years of study and more than nine years of study), and marital status (dichotomized in having a partner or without a partner). This information was self-reported by the patients in an interview and/or collected from clinical records.

Smoking and alcohol consumption were considered as lifestyle variables, which were categorized as yes and no.

### Clinical and biochemical variables

The duration of HD treatment (in <five months, ≥five months, and <12 months, and ≥12 months) (13) and the presence of associated comorbidities, such as systemic arterial hypertension (SAH) and diabetes mellitus (DM), self-reported in interviews or collected from clinical records (dichotomized as yes or no), were considered as clinical variables. The lipid profile was also evaluated, in which the following variables were considered: total cholesterol (TC), triglycerides (TG), low density lipoprotein cholesterol (LDL-c), and high density lipoprotein cholesterol (HDL-c). For the purpose of classifying the lipid profile, the criteria recommended by the National Kidney Foundation (NKF-KDOQI) (14) was considered.

### Anthropometric and body composition variables

The body mass index (BMI) was obtained by the dry weight quotient (in kg, measured using a calibrated scale, Filizola^®^, Brazil) and height (in square meters, measured by a stadiometer, Alturexata^®^, Brazil), adopting the classification proposed by World Health Organization (WHO) for adults (15). For the elderly, the classification used was proposed by Lipschitz (16). Waist circumference (WC) was measured using an inextensible measuring tape (Sanny^®^, Brazil) at the midpoint between the last rib and the iliac crest using the cut points recommended by the International Diabetes Federation (IDF) (17). All anthropometric measurements were performed after the HD session, as recommended by the National Kidney Foundation (NKF-KDOQI) (18).

Body fat percentage (%BF) was evaluated using an electrical bioimpedance (BIA) tetrapolar Biodynamics^®^ equipment 30 minutes after the dialysis session (18). Data such as weight, height, and level of physical activity were previously fed into the device and using the prediction equations of the software from the device itself, the BF was estimated. For %BF classification, %BF ≥ 25 was the threshold for men and ≥ 32 for women (19).

### Visceral adiposity index (VAI)

The VAI was calculated according to the equation proposed by Amato and cols. (6) which is gender-specific, where WC is expressed in cm, BMI in kg/m², and TG and HDL-c in mmol. The latter were obtained by multiplying the TG and HDL-c in mg/dL by 0.0113 and 0.0259, respectively (20).

Men: VAI = [WC / 39.68 + (1.88 × BMI)] × (TG / 1.03) × (1.31 / HDL-c)

Women: VAI = [WC / 36.58 + (1.89 × BMI)] × (TG / 0.81) × (1.52 / HDL-c)

The VAI was classified considering the cutoff points defined by Amato and cols. (21). For analysis purposes, the classification was dichotomized into “increased VAI levels” or “normal” VAI levels, where “increased VAI levels” refers to VAI values classified as low, moderate, and high according to age.

### Hypertriglyceridemia waist phenotype (HWP)

The HWP was defined according to the method proposed by Lemieux and cols. (5), which characterizes it from a concomitant increase in WC and TG. The cutoff points of the IDF (17) and the NKF-KDOQI (14) were adopted to classify WC and TG, respectively (≥80 cm for women and ≥90 cm for men, and TG > 150 mg/dL). The patients were grouped into “Presence” or “Absence” of HWP according to the mentioned criteria.

### Statistical analysis

The characteristics of the patients evaluated were presented in frequencies and percentages, and the proportions were compared using the Chi Square test or Fisher’s Exact test. A Poisson multivariate regression model with robust variance according to a hierarchical approach (22) was adjusted at four levels to assess the associated factors of HWP and VAI. The first level corresponded to sociodemographic variables (age, gender, and self-reported color). The second level considered the variables related to lifestyle (alcohol consumption and smoking) and the third level considered the anthropometric and body composition measurements (BMI and %BF). The fourth level was composed of clinical variables (DM, SAH, TC, HDL-c, and LDL-c).

The variables related to each level were inserted into the model simultaneously, and those with a p-value lower than 0.05 remained in the analysis at the other levels, even if the p-value was no longer significant. After the analysis of the fourth level, the variables that obtained a p-value lower than 0.05 remained in the final model. Prevalence ratios and their respective 95% confidence intervals were also estimated. The variables WC and TG did not enter the regression model for the VAI and HWP, nor did HDL-c and BMI in the regression model for the VAI, since these variables make up their respective predictive equations. In all analyses, a significance level of 5% was considered and the statistical program used was STATA 14.0 (StataCorp., College Station, TX, USA).

## RESULTS

The sample consisted of 265 patients with CKD undergoing dialysis, of which 61.13% were from São Luís city and 38.87% from Recife city. Increased VAI levels prevalence was 58.49% and that of HWP was 29.81% (Table 1). Tables 1 and 2 show other information regarding sociodemographic, lifestyle, biochemical, clinical, and body composition aspects.

**Table 1:** Sociodemographic and lifestyle characteristics of chronic kidney patients undergoing dialysis treatment at Recife-PE and São Luís-MA, 2016

Variables		n	%
		265	100
Cities			
	Recife	103	38,87
	São Luís	162	61,13
Sex			
	Men	152	57,36
	Women	113	42,64
Age group (years)			
	<40	67	25,28
	40-59	104	39,25
	≥60	94	35,47
Marital status			
	With companion	148	55,85
	No companion	117	44,15
Income (in MW)			
	<1	118	44,53
	1-4	123	46,42
	>4	24	9,06
Self-referred color			
	White	33	12,45
	No white	232	87,55
Schooling (years of study)			
	Until 9	149	56,23
	>9	116	43,77
Alcohol consumption			
	Yes	55	20,75
	No	210	79,25
Smoking			
	Yes	50	18,87
	No	146	55,09
	Ex-smoker	69	26,04

MS – Minimum salary for the year in question (R$ 880.00).

**Table 2:** Anthropometric, clinical and body composition characteristics of chronic kidney patients undergoing dialysis treatment at Recife-PE and São Luís-MA, 2016

Variables		n	%
		265	100
BMI			
	Malnutrition	42	15,85
	Eutrophy	146	55,09
	Overweight/obesity	77	29,06
WC			
	Normal	107	40,38
	High	158	59,62
HWP			
	Presence	79	29,81
	Ansence	186	70,19
VAI			
	Increased VAI levels	155	58,49
	Normal	110	41,51
%BF			
	No high	82	30,94
	High	183	69,06
DM			
	Yes	143	53,96
	No	122	46,04
SAH			
	Yes	163	61,51
	No	102	38,49
LDL-c (mg/dL)			
	<100	210	79,25
	≥100	55	20,75
TG (mg/dL)			
	<150	156	58,87
	≥150	109	41,13
HDL-c (mg/dL)			
	<40	127	47,92
	≥40	138	52,08
CT (mg/dL)			
	<200	239	90,19
	≥200	26	9,81
HD time (months)			
	<5	3	1,13
	≥5 <12	30	11,32
	≥12	232	87,55

BMI: body mass index; WC: waist circumference; HWP: hypertriglycidemic waist phenotype; VAI: visceral adiposity index; %BF: percentage of body fat; DM: diabetes; SAH: systemic arterial hypertension; LDL-c: low-density lipoprotein; TG: triglycerides; HDL-c: high density lipoprotein; CT: total cholesterol; HD time: hemodialysis time.

In the bivariate analysis according to sociodemographic and lifestyle variables (Table 3), VAI associated only with gender. Women had a greater prevalence of increased VAI levels above the cutoff points recommended by Amato and cols. (21) (p < 0.001). The HWP was associated with the variables gender, city, alcohol consumption, and smoking (p < 0.05), where patients with the presence of HWP were mostly female, living in Recife, consuming alcoholic beverages, and were smokers or ex-smokers (p < 0.05).

**Table 3:** Evaluation of the visceral adiposity index (VAI) and hypertriglyceridemia-waist phenotype (HWP) according to sociodemographic and lifestyle characteristics of patients with CKD undergoing dialysis treatment at Recife-PE and São Luís-MA, 2016

Variables		Normal VAI		Increased VAI levels		p-value	Absence HWP		Presence HWP		p-value
		n	%	n	%		n	%	n	%	
		110	41.51	155	58.49		186	70.19	79	29.81	
Cities											
	Recife	41	39.81	62	60.19	0.654	58	56.31	45	43.69	**<0.001**
	São Luís	69	42.59	93	57.41		128	79.01	34	20.99	
Sex											
	Men	77	50.66	75	49.34	<0.001	118	77.63	34	22.37	**0.002**
	Women	33	29.20	80	70.80		68	60.18	45	39.82	
Age group (years)											
	<40	30	44.78	37	55.22	0.697	48	71.64	19	28.36	0.945
	40 -59	44	42.31	60	57.69		72	69.23	32	30.77	
	≥60	36	38.30	58	61.70		66	70.21	28	29.79	
Marital status											
	With companion	62	41.89	86	58.11	0.877	107	72.30	41	27.70	0.399
	No companion	48	41.03	69	58.97		79	67.52	38	32.48	
Income (MW)											
	<1	44	37.29	74	62.71	0.456	77	65.25	41	34.75	0.244
	1-4	55	44.72	68	55.28		90	73.17	33	26.83	
	>4	11	45.83	13	54.17		19	79.17	5	20.83	
Self-referred color											
	White	15	45.45	18	54.55	0.623	23	69.70	10	30.30	0.947
	No white	95	40.95	137	59.05		166	70.26	69	29.74	
Schooling (years of study)											
	Até 9	61	40.94	88	59.06	0.831	116	71.14	43	28.86	0.701
	>9	49	42.24	67	57.76		80	68.97	36	31.03	
Alcohol consumption											
	Yes	20	36.36	35	63.64	0.384	28	50.91	27	49.09	**<0.001**
	No	90	42.86	120	57.14		158	75.24	52	24.76	
Smoking											
	Yes	21	42.00	29	58.00	0.910	26	52.00	24	48.00	**0.008**
	No	59	40.41	87	59.59		109	74.66	37	25.34	
Ex-smoker		30	43.48	39	56.52		51	73.91	18	26.09	

MW – Minimum wages for the year in question ((R$ 880.00). Statistical significance according to Chi-square or Fisher’s exact test (p < 0.05).

In the bivariate analysis, according to anthropometric, clinical and body composition characteristics (Table 4), VAI was associated with the variables %BF and TC, where patients with increased VAI levels presented such markers above the normal range (p < 0.05). The HWP, in turn, was associated with BMI, %BF, DM, SAH, and TC (p < 0.05). Most patients with HWP were overweight/obese, with a high % BF, diabetes, not hypertension and with a high CT.

**Table 4:** Evaluation of the visceral adiposity index (VAI) and hypertriglyceridemia-waist phenotype (HWP) according to anthropometric, clinical and body composition characteristics of patients with CKD undergoing dialysis treatment at Recife-PE and São Luís-MA, 2016

Variables		Normal VAI		Increased VAI levels		p-value	Absence HWP		Presence HWP		p-value
		n	%	n	%		n	%	n	%	
		110	41.51	155	58.49		186	70.19	79	29.81	
BMI											
	Malnutrition						37	88.10	5	11.90	**<0.001**
	Eutrophy						113	77.40	33	22.60	
Overweight/obesity							36	46.75	41	53.25	
%BF											
	No high	42	51.20	40	48.80	**0.032**	67	81.70	15	18.29	**0.039**
	High	68	37.16	115	62.84		119	65.03	64	34.97	
DM											
	Yes	53	37.0,6	90	62.94	0.112	87	60.84	56	39.16	**<0.001**
	No	57	46.72	65	53.28		99	81.15	23	18.85	
SAH											
	Yes	69	42.3	94	57.67	0.731	124	76.1	39	23.93	**0.008**
	No	41	40.2	61	59.8		62	60.8	40	39.22	
LDL-c (mg/dL)											
	<100	93	44.33	117	55.71	0.073	152	72.38	58	27.62	0.127
	≥100	17	30.91	38	69.09		34	61.82	21	38.18	
HDL-c (mg/dL)											
	<40						85	66.93	42	33.07	0.266
	≥40						101	73.19	37	26.81	
CT (mg/dL)											
	<200	115	43.93	134	56.07	**0.015**	175	73.23	64	26.78	**0.001**
	≥200	5	19.23	21	80.77		11	42.31	15	57.69	
HD time (months)											
	<5	2	66.70	1	33.33	0.148	2	66.70	1	33.33	0.571
	≥5 <12	8	26.70	22	73.33		19	63.30	11	36.67	
	≥12	100	43.10	132	56.90		165	71.10	67	28.88	

BMI: body mass index; HWP: hypertriglyceridemia-waist phenotype; VAI: visceral adiposity index; %BF: percentage of body fat; DM: diabetes mellitus; SAH: systemic arterial hypertension; LDL-c: low density lipoprotein cholesterol; TG: triglycerides; HDL-c: high density lipoprotein cholesterol; CT: total cholesterol; HD time: hemodialysis time. Statistical significance using the Chi-square test or Fisher’s Exact test (p < 0.05).

In the adjusted analysis, VAI was associated with females with a high %BF (p < 0.05). The HWP was associated with female gender, alcohol consumption, overweight/obesity, high %BF, and low serum HDL-c levels (p < 0.05) (Table 5).

**Table 5:** Poisson Regression Model with robust variance adjusted for risk and protection according to the visceral adiposity index (VAI) and hypertriglyceridemia-waist phenotype (HWP) in of patients with CKD undergoing dialysis treatment at Recife-PE and São Luís-MA, 2016

	VAI		PR	IC (95%)	p-value	HWP		PR	IC (95%)	p-value
**Level 1**	Sex					Sex				
		Men	1				Men	1		
		Women	**1.46**	**1.19-1.79**	**<0.001**		Women	**1.8**	**1.24- 2.62**	**0.002**
	Age group (years)					Age group (years)				
		<40	1				<40	1		
		≥40 – 59	1.02	0.78-1.32	0.880		≥40 - 59	1.04	0.65-1.66	0.849
		≥60	1.18	0.90-1.54	0.219		≥60	1.13	0.69-1.83	0.611
	Self-referred color					Self-referred color				
		No white	1				No white	1		
		White	0.92	0.67-1.27	0.655		White	1.04	0.59- 1.83	0.867
**Level 2**	Alcohol consumption					Alcohol consumption				
		No	1				No	1		
		Yes	1.09	0.85-1.39	0.475		Yes	**1.58**	**1.03-2.42**	**0.033**
	Smoking					Smoking				
		No	1				No	1		
		Yes	0.91	0.69-1.21	0.541		Yes	1.49	0.95-2.35	0.08
	Ex-smoker		1.01	0.79- 1.29	0.907	Ex-smoker		1.15	0.71-1.85	0.555
**Level 3**						BMI				
							Eutrophy	1		
							Malnutrition	0.49	0.22-1.09	0.083
							Overweight/obesity	**1.89**	**1.29-2.75**	**0.001**
	%BF					BF (%)				
		No high	1				No high	1		
		High	1.33	1.04-1.64	0.019		High	1.76	1.13- 2.75	0.012
**Level 4**	DM					DM				
		No	1				No	1		
		Yes	1.12	0.89- 1.41	0.294		Yes	1.44	0.92-2.25	0.108
	SAH					SAH				
		No	1				No	1		
		Yes	1.03	0.82-1.29	0.771		Yes	0.75	0.48- 1.10	0.138
	CT (mg/dL)					CT (mg/dL)				
		<200	1				<200	1		
		≥200	1.33	0.96-1.84	0.086		≥200	0.67	0.87-3.21	0.118
	HDL-c (mg/dL)					HDL-c (mg/dL)				
		<40					≥40	1		
		≥40					<40	**1.48**	**1.02-2.13**	**0.035**
	LDL-c (mg/dL)					LDL-c (mg/dL)				
		<100	1				<100	1		
		≥100	0.98	0.71-1.34	0.912		≥100	0.8	0.43-1.49	0.488

HWP: hypertriglyceridemia-waist phenotype; VAI: visceral adiposity index; %BF: percentage of body fat; DM: diabetes mellitus; SAH: systemic arterial hypertension; LDL-c: low density lipoprotein cholesterol; TG: triglycerides; HDL-c: high density lipoprotein cholesterol; CT: total cholesterol. Poisson Regression Model with robust variance was adjusted according to a hierarchical approach. RP: Prevalence ratio. Statistical significance according to the Poisson Regression Model p < 0.05.

## DISCUSSION

In our study, we observed a high prevalence of increased VAI levels, which is higher than the HWP prevalence. The HWP prevalence observed was similar as that reported in the literature (12) and it stood out as a marker associated with more traditional factors of CR compared to VAI. Despite the high prevalence presented, VAI represented an indicator associated with fewer variables of CR evaluated in this study, such as alcohol consumption, smoking, DM, SAH, high LDL-c, and low HDL-c, in which there was no association, even analyzing more parameters together in its predictive equation.

It is important to highlight that most participants in our study were women and although we had not evaluated issues related to menopause, they were in an age group close to this period. As demonstrated by Amrita and cols. (23), this is a phase when many hormonal variations cause imbalance in oxidative processes, since estrogen exerts beneficial effects on endothelial dysfunction, modulating the lipid profile and increasing the production of nitric oxide. As hormone levels decrease, multiple metabolic changes occur, such as reduced glucose tolerance, dyslipidemia, redox state imbalance, changes in body fat distribution, hypertension, endothelial dysfunction, and vascular inflammation, greatly contributing to increased cardiovascular risk (24).

We found that gender associated with both HWP and VAI, so that being female increases by 1.46 and 1.80 times, respectively, these conditions. In this sense, the present study is a warning to the increased risk of CVD in female HD patients with CKD.

Evaluating from another aspect, the respective equations explain in part the difference observed between HWP and increased VAI levels prevalence. The VAI is calculated according to the formula proposed by Amato and cols. (6) which considers two more variables than the equation for calculating the HWP (BMI and HDL-c). Although the majority of patients with increased VAI levels had HDL-c levels within the recommended range, we observed that the BMI prevalence in the age group of overweight/obesity was higher among patients with increased VAI levels compared to those with a VAI within the normal range (p < 0.001), which could explain the fact that increased VAI levels prevalence is higher than that presented by the HWP in our study.

Developed by Lemieux and cols. (5) the HWP derives from the hypothesis that simple variables such as WC and fasting plasma TG concentrations could be used as screening tools for the identification of the atherogenic metabolic triad. This is the first study to evaluate HWP in patients undergoing dialysis treatment in Brazil and reports a 29.81% prevalence. Previous studies, such as of Freitas and cols. (12) which evaluated 15,105 civil servants, active and retired, aged between 35 and 74 years, of both genders, from six higher education institutions located in cities in different regions of Brazil, found a 24.6% prevalence. Cabral and cols. (25) evaluated 218 patients followed up by the Hypertensive and Diabetic Registration and Monitoring System Program (HiperDia) in two health units at São Luís in Maranhão, Brazil, and found a 33% prevalence. Oliveira and cols. (26) evaluated 191 individuals from the Bahia School of Nutrition and found a 20.2% HWP prevalence. The variations found in different HWP prevalence studies may be explained due to the use of different cutoff points for WC and TG levels, as well as ethnic differences and use of lipid-lowering drugs (26).

Freitas and cols. (12), Cabral and cols. (25) and Oliveira and cols. (26) observed associations with older age, excessive alcohol consumption, ex-smoker, low HDL-c and LDL-c, TC, and C-reactive protein (PCR), fasting blood glucose ≥ 100 mg/dL or person with diabetes and presenting a greater number of CR factors. In the study by Zhe and cols. (9) with non-dialysis renal patients, the concentrations of TG, TC, HDL-c, and LDL-c in the group with HWP were significantly higher than those in the group without the phenotype. In addition, the average intima thickness of the carotid artery, one of the most accepted substitute indexes for local and generalized atherosclerosis was the highest in the group with HWP. The authors point out that HWP can be useful to predict the risk of CVD in patients with CKD. In our study, we found similar associations, showing the importance of this marker for the identification of patients with pro-atherogenic characteristics, since CVD represents the main cause of death in patients with CKD (2).

We also observed an association between high levels of %BF and the studied visceral adiposity indicators. The literature reports that adiposity, especially visceral, plays an important role in the context of CVD because it produces higher levels of inflammatory cytokines. It is associated with insulin resistance, oxidative stress markers, and inflammation (27). Thus, our findings show the importance of assessing the indicators of visceral adiposity in patients with CKD undergoing HD.

The increased VAI levels, despite its high prevalence, was an indicator associated with fewer traditional CR factors when compared to HWP. It is worth mentioning that the equation and cutoff points proposed by Amato and cols. (21) for defining and classifying the VAI were validated for a population with different characteristics than those of the Brazilian one and did not involve specific subgroups, such as patients with CKD in HD.

Amato and cols. (28) in subsequent clarifications regarding the limitations of the use of VAI, reported that the use of this index is limited to non-Caucasian populations, since the process of modeling the VAI took place in a Caucasian population aged 19 and 83 years and BMI between 20 and 30 kg/m², which may cause adverse results when applied to other populations with different characteristics. In addition, the authors emphasize that, individually or in small samples, VAI should not be applied, especially in the presence of morbid obesity (BMI > 40 kg/m²), pendular abdomen, severe hypertriglyceridemia (>279 mg/dL) and/or use of fibrates. Although only 9.81% of patients had severe hypertriglyceridemia in our sample and none had morbid obesity, we did not investigate the use of fibrates, which may be considered a limitation in this study.

International studies (7-29) have sought to assess the predictive power of VAI to discriminate CR in the HD population. However, these studies were developed in the Asian population, which have important differences between health profiles and body composition observed in Brazil. In addition, the authors used cutoff points for WC, BMI, and age, which were different from those used in our study. The aspects mentioned may not have affected the performance of HWP, as the cutoff points used in our study are similar as those used in another study with a representative sample of the Brazilian population (12).

As study limitations, as already mentioned, we could not explore and compare gender-specific associations. In addition, as this is a cross-sectional study, it is not possible to establish a causal relationship. Another important aspect is that the data analyzed in that study come from two studies in different states, which used different sampling methodologies. We also do not rule out a possible selection bias, since in Recife (PE) data was collected by convenience. In addition, as it is a sample of the population in HD specifically of certain centers restricted to São Luís and Recife cities, we recommend caution in the extrapolation of our findings. In contrast, this is the first study to evaluate HWP and VAI among the population undergoing dialysis treatment in Brazil, and it has made important contributions to the knowledge of its prevalence, hitherto unknown, in patients with CKD on HD, showing that women have a higher risk and elucidating the association of a simple and low-cost HWP indicator with important traditional CR factors.

The findings of this study show a high prevalence of increased VAI levels and a prevalence similar as that reported in the literature regarding HWP. The women evaluated are at a higher risk of having both indicators of visceral adiposity altered. The HWP, despite having a lower prevalence than increased VAI levels does and although its equation evaluates fewer parameters together, is associated with traditional CR factors. This is a practical and low-cost tool that can be used for screening CR in renal patients under dialysis treatment in primary care. We reinforce that longitudinal studies are needed to validate the analyses interpreted here.

## References

[1] Ketteler M, Block GA, Evenepoel P, Fukagawa M, Herzog CA, McCann L (2018). Diagnosis, Evaluation, Prevention, and Treatment of Chronic Kidney Disease – Mineral and Bone Disorder: Synopsis of the Kidney Disease: Improving Global Outcomes 2017 Clinical Practice Guideline Update. Ann Intern Med.

[2] Alcalde PR, Kirsztajn GM (2018). Expenses of the Brazilian Public Healthcare System with chronic kidney disease. J Bras Nefrol.

[3] Burmeister JE, Mosmann CB, Costa VB, Saraiva RT, Grandi RR, Bastos JP (2014). Prevalence of Cardiovascular Risk Factors in Hemodialysis Patients – The CORDIAL Study. Arq Bras Cardiol.

[4] Zhou C, Peng H, Yuan J, Lin X, Zha Y, Chen H (2018). Visceral, general, abdominal adiposity and atherogenic index of plasma in relatively lean hemodialysis patients. BMC Nephrol.

[5] Lemieux I, Pascot A, Couillard C, Lamarche B, Tchernof A, Alméras N (2000). Hypertriglyceridemic Waist t: a marker of the atherogenic metabolic triad (hyperinsulinemia; hyperapolipoprotein B; small, dense LDL) in men?. Circulation.

[6] Amato MC, Giordano C, Galia M, Criscimanna A, Vitabile S, Midiri M (2010). Visceral Adiposity Index: A reliable indicator of visceral fat function associated with cardiometabolic risk. Diabetes Care.

[7] Chen HY, Chiu YL, Chuang YF, Hsu SP, Pai MF, Yang JY (2014). Visceral adiposity index and risks of cardiovascular events and mortality in prevalent hemodialysis patients. Cardiovasc Diabetol.

[8] Zhou C, Zhan L, Yuan J, Tong X, Peng Y, Zha Y (2019). Comparison of visceral, general and central obesity indices in the prediction of metabolic syndrome in maintenance hemodialysis patients. Eat Weight Disord.

[9] Zhe X, Bai Y, Cheng Y, Xiao H, Wang D, Wu Y (2012). Hypertriglyceridemic Waist is Associated with Increased Carotid Atherosclerosis in Chronic Kidney Disease Patients. Nephron Clin Pract.

[10] Costa J, Pinho CPS, Maio R, Diniz A da S, Carvalho de TR, Barboza YACO (2020). Adequação dialítica e estado nutricional de indivíduos em hemodiálise. BJD.

[11] Hortegal EVF, Alves JJDA, Santos EJF, Nunes LCR, Galvão JC, Nunes RF (2020). Sarcopenia and inflammation in patients undergoing hemodialysis. Nutr Hosp.

[12] Freitas RS, Fonseca da MJM, Schmidt MI, Molina MCB, Almeida de MCC (2018). Fenótipo cintura hipertrigliceridêmica: fatores associados e comparação com outros indicadores de risco cardiovascular e metabólico no ELSA-Brasil. Cad Saúde Pública.

[13] Chang TI, Ngo V, Streja E, Chou JA, Tortorici AR, Kim TH (2017). Association of body weight changes with mortality in incident hemodialysis patients. Nephrol Dial Transplant.

[14] K/DOQ Group (2003). Introduction. Am J Kidney Dis.

[15] World Health Organization (WHO) (1998). WHO Consultation on Obesity.

[16] Lipschitz DA (1994). Screening for nutritional status in the elderly. Prim Care.

[17] Alberti KG, Zimmet P, Shaw J (2006). Metabolic syndrome-a new world-wide definition. A Consensus Statement from the International Diabetes Federation. Diabet Med.

[18] National Kidney Foundation Guideline on Nutrition in CKD. Clinical practice guideline for nutrition in chronic kidney disease: 2019 update.

[19] Lohman TG (1993). Advances in body composition assessment. Cad Saúde Pública.

[20] Notice (2013). Kidney International Supplements.

[21] Amato MC, Giordano C, Pitrone M, Galluzzo A (2011). Cut-off points of the visceral adiposity index (VAI) identifying a visceral adipose dysfunction associated with cardiometabolic risk in a Caucasian Sicilian population. Lipids Health Dis.

[22] Fuchs SC, Victora CG, Fachel J (1996). Modelo hierarquizado: uma proposta de modelagem aplicada à investigação de fatores de risco para diarreia grave. Rev Saúde Pública.

[23] Amrita J, Mahajan M, Bhanwer AJS, Mohan G (2016). Oxidative Stress: An Effective Prognostic Tool for an Early Detection of Cardiovascular Disease in Menopausal Women. Biochem Res Int.

[24] Klisic A, Kavaric N, Vujcic S, Spasojevic-Kalimanovska V, Kotur-Stevuljevic J, Ninic A (2020). Total oxidant status and oxidative stress index as indicators of increased Reynolds risk score in postmenopausal women. Eur Rev Med Pharmacol Sci.

[25] Cabral NAL, Ribeiro VS, da Cunha França AKT, Salgado JVL, dos Santos AM (2012). Cintura hipertrigliceridêmica e risco cardiometabólico em mulheres hipertensas. Rev Assoc Méd Bras.

[26] Cunha de Oliveira C (2014). Fenotipo Cintura Hipertrigliceridémica: Relación entre cambios. Nutr Hosp.

[27] Yajima T, Arao M, Yajima K, Takahashi H, Yasuda K (2019). The associations of fat tissue and muscle mass indices with all-cause mortality in patients undergoing hemodialysis. PLoS One.

[28] Amato MC, Giordano C (2014). Visceral adiposity index: an indicator of adipose tissue dysfunction. Int J Endocrinol.

[29] El Said HW, Mohamed OM, El Said TW, El Serwi AB (2017). Central obesity and risks of cardiovascular events and mortality in prevalent hemodialysis patients. Int Urol Nephrol.

